# Dataset-Dependent Performance of Active Learning Acquisition Functions for Multi-Objective Materials Optimization

**DOI:** 10.3390/ma19214736

**Published:** 2026-11-07

**Authors:** Jingjing Yan, Qing Zhao, Huiling He, Zhiqiang Mu, Haoyu Song, Yiming Chen, Xiaobing Hu, Jincheng Wang

**Affiliations:** 1 School of Architecture, Kashi University, Kashi 844000, China; 2 School of Civil Engineering, Kashi University, Kashi 844000, China; 3 Xi’an Rare Metal Materials Institute Co., Ltd., Xi’an 710016, China; 4 State Key Laboratory of Solidification Processing, Northwestern Polytechnical University, Xi’an 710072, China; 5 School of Metallurgical Engineering, National and Local Joint Engineering Research Center for Functional Materials Processing, Xi’an University of Architecture and Technology, Xi’an 710055, China

**Keywords:** active learning, acquisition functions, multi-objective optimization

## Abstract

Active learning (AL) promises accelerated multi-objective materials discovery, yet acquisition-function choice is often made without matching dataset, budget, and target metric. We retrospectively compared EI-random, EI-cent, EI-maxmin, and EI-prod on Al alloy, steel, and Cu alloy datasets across 100 randomized cold-start trials, using hypervolume, true Pareto-sample discovery, inverted generational distance, balanced-performance score, and iterations to 95% target. EI-prod is best overall on Al dataset; EI-maxmin improves Pareto-front recovery and convergence on steel and Cu datasets; EI-cent gives limited, inconsistent gains. No strategy dominates all datasets or budgets. These results provide a metric-aware, outcome-specific selection guide: use EI-prod for early balanced candidates, and EI-maxmin for broader Pareto-front exploration. They also warn that similar balanced performance does not ensure equivalent front reconstruction and that dataset-specific rankings should inform, not dictate, AL design in materials screening. This reframes acquisition selection as a decision-making, not universal-ranking, problem.

## 1. Introduction

The development of novel materials with tailored properties is important for applications in architecture, aerospace, energy, environment, and healthcare [1,2,3,4,5]. Traditional materials discovery relies heavily on trial-and-error synthesis and characterization, making the process time-consuming and costly. This motivates methods that can reduce the number of synthesis and characterization cycles. Rapid advances in artificial intelligence (AI) have enabled data-driven approaches to materials discovery [6,7,8,9]. Data-driven methods can improve the efficiency of candidate screening and experimental decision-making, but their predictions still require validation through experimental characterization and physical modeling. Experimental characterization can reveal the microstructure, molecular interactions, and deformation mechanisms underlying changes in material properties, thereby helping to determine whether the correlations identified by machine learning models have a physical basis [10,11]. Therefore, the integration of computational modeling, experimental characterization, and iterative experiments is an important pathway for transforming data-driven screening into a reliable materials design strategy. Nevertheless, a gap remains between computational predictions and experimental validation. Closing this gap requires iterative selection, measurement, and model updating rather than offline prediction alone.

Active learning (AL) provides one framework for this iterative interaction between AI and experiment [12,13,14]. Unlike passive learning, in which models are trained on static datasets, AL iteratively selects the most informative candidates using predictive uncertainty, expected improvement, or other acquisition criteria for validation through subsequent experiments [15,16,17]. By prioritizing high-value experiments, AL can reduce the number of evaluations required to identify materials with desirable properties. Accordingly, AL has been applied in diverse applications, including the discovery of high-entropy alloys [18,19], optimization of polymer synthesis conditions [20,21,22], and screening of effective catalysts [13,23,24]. These applications motivate a closer examination of how candidate-selection rules affect multi-objective search.

AL research has produced a range of acquisition functions, also referred to as utility functions. Despite this growing diversity, several established selection rules remain common, including random sampling as a baseline (EI-random) [25,26,27], centroid-based acquisition (EI-cent) [17], max-min-distance-driven exploration (EI-maxmin) [16,28], and the multi-objective expected improvement product (EI-prod) [29,30,31,32]. Conceptually, the performance of an acquisition function can be characterized along several fundamental dimensions: its ability to expand coverage of the objective space, identify the true Pareto-optimal boundary, accelerate convergence toward the Pareto front, and improve overall multi-objective optimization performance. However, these characteristics are often conflated or evaluated ambiguously in previous studies [33,34,35,36,37]. Existing work commonly relies on a single metric or a one-dimensional assessment, making it difficult to disentangle the distinct contributions of an acquisition strategy to exploration, boundary recovery, convergence, and sample efficiency [34,37,38,39,40]. Consequently, the specific strengths and limitations of different acquisition functions—and, in particular, the outcomes favored by each function—remain insufficiently understood. A comparison across complementary metrics and repeated initializations can make strategy selection more transparent.

To clarify these differences, we compare four representative acquisition strategies across three materials datasets using complementary measures of objective-space coverage, recovery and representation of the true Pareto front, and discovery of a balanced high-performance candidate. Repeated cold-start simulations allow us to examine both average trajectories and variation across initial labeled sets. Rather than treating one metric as a proxy for overall success, we ask which strategies favor rapid joint improvement and which support more complete exploration of trade-off solutions. The datasets, acquisition functions, and evaluation protocol are described below.

The present study is a retrospective benchmark based on finite datasets in which the objective values of all candidates are already available. This setting allows the true Pareto set and the final performance of every selected candidate to be determined, but it does not reproduce all conditions of a prospective experimental campaign. In practice, candidate synthesis may fail, measurements may contain experimental noise, some candidates may be unavailable or difficult to manufacture, and the costs and durations of different experiments may vary substantially. Therefore, the rankings reported here should be interpreted as comparative evidence under a fixed candidate pool and evaluation protocol. Their transfer to prospective materials optimization requires additional consideration of measurement uncertainty, candidate feasibility, experimental cost, and model extrapolation.

## 2. Methodology

### 2.1. Dataset Collection and Preprocessing

Three datasets were collected: an aluminum (Al) alloy dataset (N=1043) [41], a steel dataset (N=437) from the National Institute for Materials Science database (NIMS structural materials databases and cross search engine—MatNavi. VTT Symp), and a copper (Cu) alloy dataset (N=190) from our experiments. For each dataset, the material performance objectives may have different directions: some objectives are maximized, whereas others are minimized. To unify the optimization direction, all objectives were converted into maximization problems. Because different objectives have different numerical scales, min-max normalization was applied within each dataset to map all objective values to [0,1]:

$$y_{m}^{norm}=\frac{y_{m}-y_{m}^{min}}{y_{m}^{max}-y_{m}^{min}}$$

where $y_{m}^{min}$ and $y_{m}^{max}$ are the minimum and maximum values of objective m in the full dataset. After these transformations, all objectives lie in [0,1] and are to be maximized.

In this retrospective benchmark, the minimum and maximum values of each objective were calculated from the complete dataset to define a fixed, common normalized objective space. The normalized values were used for SVR training, acquisition calculations, and performance evaluation, with the same scaling applied to all strategies within each dataset. This convention facilitates comparison under a consistent objective-space scale, but uses information about objective ranges that would not generally be available at the start of a prospective campaign. Since objective scaling can influence surrogate fitting and acquisition values, the reported strategy rankings are conditional on this normalization convention. Their sensitivity to scaling based only on sequentially available information was not evaluated here. For prospective applications, scaling should be based on predefined physical ranges, independent reference data, or the currently labeled samples, with its influence on acquisition behavior assessed accordingly.

For each dataset, the reference Pareto-optimal set, denoted by $P_{true}$, was computed from the recorded objective values of all valid samples and held fixed during the retrospective simulations. Here, “true” denotes the dataset-wide reference relative to the currently discovered front; it does not imply a noise-free physical Pareto set. The recorded values were treated as fixed observations, without additional measurement-noise perturbations. For maximization problems, point a dominates point b if and only if $a_{m}\geq b_{m},\;\forall \;m$ and at least one inequality is strict. All points that are not dominated by any other point constitute the Pareto-optimal set. As listed in Table 1, the detailed information related to the three datasets has been clearly provided.

All three datasets involve two simultaneous maximization objectives. The Al dataset has a weak overall objective correlation, whereas the steel and Cu datasets show strong negative correlations between their objectives. The corresponding true Pareto sets contain 14, 19, and 20 samples for Al, steel, and Cu, respectively. After min–max normalization, the estimated Pareto-front lengths are 1.109, 1.337, and 1.245, respectively. Here, the objective correlations were calculated using all valid records before active-learning simulation. The Pareto-front length was calculated after min–max normalization of the two objectives and ordering the Pareto-optimal points by the first objective. These descriptors provide context for interpreting the dataset-dependent results. Because sample size, material composition, objective definitions, and front geometry vary together across the three datasets, they do not isolate the contribution of front geometry to acquisition performance. The reported front characteristics are therefore used for descriptive interpretation rather than as evidence of a causal relationship.

For each dataset, let N denote the total number of samples. The initial training set size was set as $n_{0}=\max\left\{10,\;[0.02N]\right\}$, where [·] denotes the ceiling operator. And the remaining $N-n_{0}$ samples were used as the candidate pool U. We note that 2% is regarded as an empirical setting that balances initial model trainability and candidate pool size, rather than as a universally optimal ratio. More importantly, such a small ratio reflects the fact that real material datasets are extremely sparse relative to the vast unexplored space. Accordingly, for the Al dataset, $n_{0}=20$ and |U|=1023; for the steel dataset, $n_{0}=10$ and |U|=427; and for the Cu alloy dataset, $n_{0}=10$ and |U|=180. This preprocessing ensures that all objectives are consistently oriented and comparable, and that the initial training set is sufficiently large while retaining a substantial candidate pool for subsequent active learning.

It should be noted that the Cu alloys were subjected to the following thermomechanical treatment sequence: homogenization at 1000 °C for 2 h, hot rolling with a 70% thickness reduction, solution treatment at 980 °C for 2 h, cold rolling with an 80% reduction, and peak-aging at 450 °C. Tensile tests were conducted on a SUNS UTS 5105 universal testing machine at room temperature with a loading rate of 0.5 mm/min. The electrical conductivity (EC) values of the alloy samples were measured using a D60k digital eddy current metal conductivity meter.

### 2.2. Machine Learning Modeling and Active Learning

The SVR models used the numerical input descriptors listed in Table 1, comprising 16 descriptors for Al, 15 for steel, and 10 for Cu. Target properties were excluded from the input features. No categorical encoding, feature selection, or dimensionality reduction was applied. At each active-learning iteration, a StandardScaler was fitted exclusively to the currently labeled input samples, and the resulting transformation was applied to both the labeled samples and the candidate pool. Thus, the feature-wise means and standard deviations used for input standardization were updated from the labeled set only. This input-standardization procedure is distinct from the full-dataset normalization of the target properties described in Section 2.1.

To guide the sequential selection of informative samples, a bagging-based support vector regression (SVR) framework was adopted to provide both predictive means and predictive uncertainties for each material performance objective. Specifically, for each objective m, an ensemble of $N_{B}=\;100$ SVR sub-models was constructed via bootstrap resampling. Each base SVR used a radial basis function (RBF) kernel with C = 10, ϵ = 0.01, and γ = scale. The standard deviation across bootstrap SVR predictions was used as a practical proxy for predictive uncertainty in the acquisition calculations. Together with the ensemble mean, it defines the approximate Gaussian predictive distribution adopted for evaluating EI and PI. This ensemble spread reflects disagreement among the base estimators and is not assumed to constitute a fully calibrated predictive uncertainty. No separate uncertainty-calibration procedure was applied. The resulting acquisition values are therefore interpreted within the adopted predictive approximation, with the same modeling procedure used across the acquisition strategies.

For a candidate sample $x_{i}$, the predictions from the $N_{B}$ sub-models for objective m are denoted as

$$f_{1,m}\left(x_{i}\right),\;f_{2,m}\left(x_{i}\right),\cdots ,f_{N_{B},m}\left(x_{i}\right)$$

The predictive mean $\mu_{m}\left(x_{i}\right)$ and predictive standard deviation $\sigma_{m}\left(x_{i}\right)$ are then computed as

$$\mu_{m}\left(x_{i}\right)=\frac{1}{N_{B}}\sum_{n=1}^{N_{B}}f_{n,m}\left(x_{i}\right)$$

$$\sigma_{m}\left(x_{i}\right)=std\left(f_{1,m}\left(x_{i}\right),\;f_{2,m}\left(x_{i}\right),\cdots ,f_{N_{B},m}\left(x_{i}\right)\right)$$

where $std\left(\cdot \right)$ denotes the standard deviation operator. These quantities, $\mu_{m}\left(x_{i}\right)$ and $\sigma_{m}\left(x_{i}\right)$, are subsequently used as inputs to compute an acquisition function, including expected improvement (EI), whose detailed formulation is provided in the following section.

The active learning procedure was performed for each dataset and each acquisition function according to the following steps:

(1) Initialization. Randomly select $n_{0}$ samples from the full dataset to form the initial training set $L_{0}$, where $n_{0}=\max\left\{10,\;0.02N\right\}$. The remaining samples constitute the candidate pool U.
(2) Iterative loop. For t=1,2,…,T:
  Train the bagging SVR models for all objectives using the current training set $L_{t-1}$.
  Compute the current Pareto front $P_{t-1}$ based on the true objective values of the samples in $L_{t-1}$.
  For each candidate sample x in U, compute the acquisition function value U(x) using the predictive means and uncertainties from the bagging SVR models.
  Select the sample $x^{*}$ that maximizes the acquisition function: $x^{*}\underset{x\in U}{\arg\max}U(x)$.
  Remove $x^{*}$ from the candidate pool and add it to the training set: $L_{t}=L_{t-1}⋃\left\{x^{*}\right\}$.
  Record the current performance metrics.
(3) Stopping criterion. Repeat the loop until the candidate pool is empty or a predefined experimental budget is reached. In this work, one sample was selected per iteration, i.e., the batch size was set to 1.

The maximum acquisition budgets were set to 200, 100, and 50 iterations for the Al, steel, and Cu datasets, respectively, with one sample acquired per iteration. These limits correspond to approximately 20–30% of the initial candidate pools. They were selected as extended retrospective evaluation horizons rather than as recommended lengths for prospective experimental campaigns. In the high-cost, long-duration synthesis and characterization settings considered here, conducting 50 or more sequential experimental rounds would often be impractical. The selected limits therefore extend beyond the short experimental campaigns motivating this study and allow the evolution of acquisition performance to be examined over a wider budget range. Nevertheless, the budgets do not represent identical sampling fractions across datasets. Cross-dataset comparisons are therefore descriptive and do not isolate dataset effects from differences in the evaluated sampling fraction.

For each dataset, 100 randomized trials were performed. Within each trial, all four acquisition strategies shared exactly the same initial labeled set and candidate pool, enabling paired comparisons under a common initialization. Different trials used independently randomized initial sets. Initialization was controlled using the seed 20,260,817+r, where r=0,⋯,99. Separate reproducible random streams were derived from the trial index, dataset identifier, and strategy identifier for bootstrap resampling, random acquisition, and numerical tie-breaking. The bootstrap random states were additionally indexed by the acquisition iteration and objective. Thus, initialization was shared across strategies, whereas subsequent bootstrap realizations were strategy-specific and reproducible. PI was evaluated analytically, so no Monte Carlo random seed was required.

This iterative procedure integrates predictive uncertainty into the sample selection process to update the model and the approximation of the observed Pareto front. Additionally, the implementation was conducted in Python 3.12.10 using the scikit-learn machine-learning library by the Integrated Development Environment of Spyder.

### 2.3. Definition of Acquisition Functions

Let $L_{t}$ denote the currently labeled training set, and let $P_{t}$ be the Pareto front formed by the true objective values of the samples in $L_{t}$. For each candidate sample x, the bagging SVR models provide the predictive mean vector $\mu \left(x\right)=\left(\mu_{1}\left(x\right),\cdots \mu_{M}\left(x\right)\right)$ and the predictive standard deviation vector $\sigma \left(x\right)=\left(\sigma_{1}\left(x\right),\cdots \sigma_{M}\left(x\right)\right)$. Four utility functions were considered, leading to four active learning strategies: EI-prod, EI-cent, EI-maxmin, and EI-random.

Expected hypervolume improvement (EHVI) is an established acquisition criterion that directly targets the expected increase in dominated hypervolume. The present benchmark focuses on four contrasting strategies: a product of marginal expected improvements, two probability-of-improvement criteria combined with geometric distance terms, and random sampling. This selection allows their dataset- and budget-dependent behavior to be examined within a common surrogate-model framework, but does not constitute a comprehensive comparison of multi-objective acquisition functions. In particular, using HV as an evaluation metric does not replace an EHVI acquisition benchmark. The reported advantages are therefore restricted to comparisons among the four investigated strategies. Including EHVI would be a valuable extension for assessing whether these observations persist across a broader set of acquisition criteria.

In this paper, the prefix “EI” is used as an umbrella label for the four acquisition strategies included in the comparison, rather than as a claim that all four strategies are mathematically expected-improvement estimators. EI-prod is the product of single-objective expected improvements, whereas EI-cent and EI-maxmin combine a probability-of-improvement term with a centroid-distance or maximin-distance term, respectively. EI-random is a random baseline and does not calculate an expected-improvement quantity. The mathematical definitions in Section 2.3.1, Section 2.3.2, Section 2.3.3 and Section 2.3.4 determine the precise meaning of each strategy. The existing strategy names are retained throughout the text and figures to maintain consistency with the computational implementation and reported results.

#### 2.3.1. Utility Function 1: Product of Single-Objective Expected Improvements (EI-Prod)

For a single objective m, the expected improvement (EI) [42,43] is defined as

$${EI}_{m}\left(x\right)=E\left[\max\left({\hat{f}}_{m}\left(x\right)-f_{m}^{*},\;0\right)\right]$$

where ${\hat{f}}_{m}\left(x\right)\sim N\left(\mu_{m}\left(x\right),\sigma_{m}\left(x\right)\right)$, and $f_{m}^{*}$ is the best observed value of objective m in the current training set $L_{t}$. Under the Gaussian assumption, the analytical expression is

$${EI}_{m}\left(x\right)=\sigma_{m}\left(x\right)\left[z_{m}\Phi \left(z_{m}\right)+\phi \left(z_{m}\right)\right]$$

$$z_{m}=\frac{\mu_{m}\left(x\right)-f_{m}^{*}}{\sigma_{m}\left(x\right)}$$

where Φ and ϕ denote the standard normal cumulative distribution function and probability density function, respectively.

The multi-objective utility function is defined as the product of the single-objective EI values [29,30,31,32]:

$$U_{EI-prod}\left(x\right)=\prod_{m=1}^{M}{EI}_{m}\left(x\right)$$

EI-prod is defined here as a product of marginal EI scores. It does not explicitly model cross-objective predictive covariance and is not interpreted as a covariance-aware joint expected improvement.

In the implementation, the predictive standard deviation for each objective was bounded below by 10^−12^ before evaluating the Gaussian EI expression to avoid division by zero. Each calculated EI value was then clipped to be non-negative. The multi-objective acquisition score was computed directly as the product of the single-objective EI values; logarithmic evaluation was not used. No positive numerical floor was imposed on the EI values themselves. Consequently, if an EI value was numerically zero for either objective, the corresponding product score was zero. This implementation avoids undefined logarithms, although extremely small EI values may still underflow to zero in floating-point arithmetic.

#### 2.3.2. Utility Function 2: Probability of Improvement × Centroid Distance (EI-Cent)

First, the multi-objective probability of improvement $PI\left(x\right)$ is computed as the probability that the candidate point x is not dominated by the current Pareto front $P_{t}$. For computational tractability, the joint predictive distribution was approximated by the product of the marginal Gaussian distributions:

$$p(y|x)\approx \prod_{m-1}^{M}N\left(y_{m};\mu_{m}\left(x\right),\sigma_{m}^{2}\left(x\right)\right)$$

Accordingly, the multi-objective probability of improvement $PI\left(x\right)$ was calculated by integrating this approximated joint density over the non-dominated region Ω. The factorization concerns the predictive distribution at a given candidate and does not imply physical independence between the material properties. Although the steel and Cu datasets exhibit pronounced objective trade-offs, correlation between measured objectives across a dataset is not equivalent to the conditional predictive covariance at an individual candidate.

Neglecting predictive covariance may affect the estimated probability of entering the non-dominated region and, consequently, candidate rankings. The magnitude and direction of this effect depend on the predictive dependence structure and the current Pareto-front geometry. The present comparisons use the factorized approximation throughout; their sensitivity to covariance-aware predictive modeling was not evaluated. Accordingly, the observed rankings are interpreted within this modeling framework.

Because the present study involves two objectives, the integral was evaluated analytically by decomposing the non-dominated region into piecewise intervals defined by the currently observed Pareto front. After sorting the Pareto-front points according to the first objective, the probability contribution of each interval was calculated using the cumulative distribution function of the corresponding marginal Gaussian distributions and then summed over all intervals. Thus, no Monte Carlo sampling was used for the evaluation of the multi-objective PI, and no Monte Carlo random seed or convergence criterion was required. For each candidate, the computational cost scales with the number of points on the current Pareto front.

Next, the centroid of the current Pareto front $P_{t}=\left\{p_{1},p_{2},\cdots ,p_{K}\right\}$ is computed as

$$c\left(P_{t}\right)=\frac{1}{K}\sum_{k=1}^{K}p_{k}$$

The Euclidean distance between the candidate predictive mean and the centroid is

$$D_{centroid}\left(x\right)=\left\|\mu \left(x\right)-c\left(P_{t}\right)\right\|$$

The final utility function [15,16,17] is then

$$U_{PI-cent}\left(x\right)=PI\left(x\right)\cdot D_{centroid}\left(x\right)$$

#### 2.3.3. Utility Function 3: Probability of Improvement × Maximin Distance (EI-Maxmin)

As in Section 2.3.2, the multi-objective probability of improvement was evaluated using the same analytical piecewise integration procedure for the two-objective case. The resulting PI values were then multiplied by the maximin-distance term. As in Section 2.3.2, $PI\left(x\right)$ was calculated using the marginal-Gaussian approximation and the independence assumption described above. Therefore, EI-maxmin inherits the same approximation concerning the joint distribution of the objectives.

Next, the maximin distance is defined as the distance from the candidate point to the nearest point in the current Pareto front [15,16,17]:

$$D_{maxmin}\left(x\right)=\underset{p\in P_{t}}{\max}{\left[\min\left\{\mu_{1}\left(x\right)-p_{1},\mu_{2}\left(x\right)-p_{2}\right\}\right]}_{+}$$

where ${\left[z\right]}_{+}=max(z,0)$. No random positive perturbation was added to the distance term. Therefore, a candidate whose predicted improvement relative to the current Pareto front is zero retains a distance value of zero.

$$U_{PI-maxmin}\left(x\right)=PI\left(x\right)\cdot D_{maxmin}\left(x\right)$$

After the acquisition utility had been calculated, a very small random term $10^{-8}\mu \left(x\right)$, where $\mu \left(x\right)\sim U(0,1)$, was added only to break exact ties between candidates with identical utility values. This tie-breaking term was generated independently for each candidate and iteration and was controlled by the trial-specific random seed. Its magnitude is negligible compared with non-identical utility differences and therefore does not change the ranking of non-tied candidates.

#### 2.3.4. Utility Function 4: Random Baseline (EI-Random)

As a baseline strategy, random sampling is defined as

$$U_{random}\left(x\right)=random\left(x\right)$$

where $random\left(x\right)$ denotes a random value assigned to each candidate sample.

The four active learning strategies are denoted as EI-prod, EI-cent, EI-maxmin, and EI-random, respectively, corresponding to the utility functions defined above. Notably, although the last strategy is denoted as EI-random for consistency with the comparison framework, it represents a purely random sampling baseline and does not contain an EI or PI calculation.

### 2.4. Performance Evaluation Metrics

To evaluate the effectiveness of the active learning strategies, three front-level metrics and one time-to-target metric were employed: hypervolume (HV), discovery rate of true Pareto-optimal samples (DR), inverted generational distance (IGD), and a threshold-based metric $N_{0.95-MP}$ derived from the maximum product of normalized objective values.

#### 2.4.1. Hypervolume (HV)

For the current labeled training set $L_{t}$, the hypervolume is computed with respect to a reference point r:

$$HV\left(L_{t};r\right)=⋀\left(\underset{s\in L_{t}}{⋃}\left[r_{1},s_{1}\right]\times \left[r_{2},s_{2}\right]\times \cdots \times \left[r_{M},s_{M}\right]\right)$$

where $⋀\left(\cdot \right)$ denotes the Lebesgue measure, and $s\;=\;\left(s_{1},s_{2},\cdots ,\;s_{M}\right)$ is the true objective vector of sample s. Since all objective values have been normalized to [0,1] and converted to maximization, the reference point can be set to r=(0,0,…,0). A larger HV indicates that the discovered set dominates a greater volume of objective space.

#### 2.4.2. Discovery Rate of True Pareto-Optimal Samples (DR)

The discovery rate (DR) measures the fraction of the true Pareto-optimal set that has been found in the current labeled set:

$$DR\left(L_{t}\right)=\frac{\left|L_{t}⋂P_{true}\right|}{\left|P_{true}\right|}$$

Here, $P_{true}$ is the fixed reference Pareto set defined from the recorded objective values, and $L_{t}⋂P_{true}$ contains its members already included in the labeled set. A higher DR therefore indicates greater recovery of this empirical reference set. Measurement uncertainty may change the dominance relationships of candidates with closely spaced objective values and hence their reference-set membership. DR is consequently interpreted as a dataset-based discovery measure, rather than a probability that a candidate remains Pareto-optimal under repeated experimental measurements.

#### 2.4.3. Inverted Generational Distance (IGD)

The inverted generational distance (IGD) measures the average distance from each point in the true Pareto front to the nearest point in the current labeled set:

$$IGD\left(L_{t}\right)=\frac{1}{\left|P_{true}\right|}\sum_{p\in P_{true}}\underset{q\in L_{t}}{\min}\left\|p-q\right\|$$

A smaller IGD indicates that the current labeled set is closer to the true Pareto front, indicating better proximity to and coverage of the reference front.

#### 2.4.4. Threshold-Based Metric $N_{0.95-MP}$

To further assess the ability of the active learning strategies to identify samples with high overall performance, a threshold metric ($N_{0.95-MP}$) based on the maximum product of normalized objective values was introduced. Because some trials did not reach the 95% target within the maximum acquisition budget, target attainment was treated as a time-to-event outcome with right censoring. We therefore report two complementary quantities: (i) the target attainment rate, defined as the fraction of trials that reached the target within the prescribed budget, and (ii) the mean target-reaching budget calculated among successful trials only. Unsuccessful trials were not assigned an observed target-reaching time and were not treated as completed events.

For the current labeled set $L_{t}$, the maximum product (MP) is defined as

$$MP\left(L_{t}\right)=\underset{s\in L_{t}}{\max}\prod_{m=1}^{M}y_{s,m}^{norm}$$

where $y_{s,m}^{norm}$ is the normalized value of objective m for sample s, ranging in [0,1] and to be maximized. When the number of objectives is large and the product tends to become extremely small, the geometric mean is recommended for numerical stability:

$$MP\left(L_{t}\right)=\underset{s\in L_{t}}{\max}{\left(\prod_{m=1}^{M}y_{s,m}^{norm}\right)}^{1/M}$$

The geometric mean is used in this work because it preserves the ranking by the product for a fixed number of objectives and improves numerical interpretability.

The geometric-mean-based MP is used only as an evaluation metric and is not identical to the EI-prod acquisition function. EI-prod multiplies predicted single-objective expected improvements, whereas MP is calculated from the normalized measured objectives of the labeled samples. Therefore, any similarity between the EI-prod and MP rankings should be interpreted as a possible alignment of optimization preference rather than mathematical equivalence.

Let ${MP}_{global}$ denote the maximum normalized product (geometric mean) over the entire dataset. Let $K_{0.95-MP}$ denote the first acquisition round at which the current MP reaches at least 95% of its dataset-wide reference value:

$$K_{0.95-MP}=\min\left\{k\in \left\{0,\cdots ,K_{max}\right\}:MP\left(L_{t}\right)\geq 0.95\cdot {MP}_{global}\right\}$$

where $L_{t}$ contains $n_{0}+k$ labeled samples and $K_{max}$ is the maximum number of acquisitions after initialization. This threshold of 95% provides an operational analogue of a practical materials-design task: identifying a candidate with a near-best combination of properties within a limited experimental budget, rather than necessarily locating the exact best candidate in the reference dataset. The product criterion favors simultaneously high normalized objective values and penalizes a low value in either objective. However, 95% of the reference product is a benchmarking convention, rather than an application-specific acceptance criterion, and does not require each individual property to reach 95% of its maximum. In prospective materials design, the stopping target should instead reflect the required property levels and acceptable trade-offs of the intended application.

The corresponding total labeled-sample budget is

$$N_{0.95-MP}=n_{0}+K_{0.95-MP}$$

Therefore, $K_{0.95-MP}$ excludes the initial training samples, whereas $N_{0.95-MP}$ includes them. The maximum total labeled-sample budget is $B=n_{0}+K_{max}$.

## 3. Results and Discussion

We compared the four strategies on the Al, steel, and Cu datasets using hypervolume (HV), Pareto-optimal sample discovery rate (DR), inverted generational distance (IGD), and maximum balanced performance (MP). These metrics distinguish dominated-space expansion, recovery of individual Pareto-optimal samples, representation of the full front, and discovery of a single well-balanced candidate. Results from 100 randomized trials are summarized by mean trajectories and distributions at selected iterations. Within each dataset, the four strategies were paired through their shared initial labeled sets, whereas different trials used independently randomized initializations. The reported run-to-run variation therefore reflects variation in initialization together with the controlled stochastic components of the algorithms. We also report the sampling budget required to reach 95% of the reference MP. The shared initial labeled sets provide a matched experimental design. The reported summaries describe strategy-specific mean trajectories and distributions; paired differences and their confidence intervals were not estimated. Accordingly, comparisons of sampling efficiency refer to observed budget differences under the evaluated conditions.

Table 2 provides a numerical summary of HV, DR, and IGD at selected fixed budgets. Values are reported as the mean ± one sample standard deviation across 100 paired runs. The four strategies are compared at the same budget within each dataset. These summaries describe observed performance and variability and do not establish statistical significance.

The geometric characteristics of the reference datasets provide useful context for interpreting the strategy rankings. The Al objectives show only a weak overall correlation (*r* = −0.072) and their reference Pareto set contains 14 samples. In contrast, steel and Cu exhibit stronger negative objective correlations (*r* = −0.873) and (*r* = −0.711), respectively) and contain larger reference Pareto sets of 19 and 20 samples. Their normalized Pareto fronts are also longer than those of Al. These observations are consistent with a more pronounced trade-off and a greater need to explore separated regions of the objective space in the steel and Cu datasets. However, because sample size, descriptor dimension, objective definitions, and front geometry vary simultaneously, these geometric quantities provide descriptive context rather than an isolated causal explanation for the observed acquisition-function rankings.

### 3.1. Pareto-Front Expansion and Hypervolume Improvement

**Figure 1:**
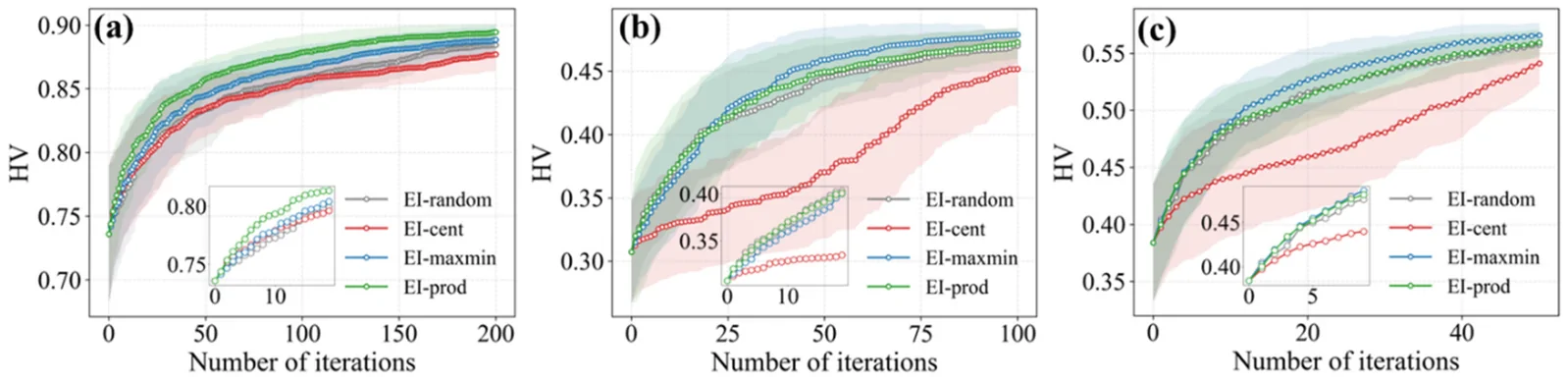
Evolution of HV with the number of active-learning iterations for the (**a**) Al alloy, (**b**) steel, and (**c**) Cu alloy datasets. The four curves correspond to EI-random, EI-cent, EI-maxmin, and EI-prod. Insets enlarge the initial optimization stage, and each curve represents the mean across 100 paired runs, and the shaded color-band indicates standard deviation across these runs. Within each dataset, the four strategies share the same initial labeled set in each paired run. Higher HV indicates greater coverage of dominated objective space. The horizontal axis counts acquisitions after initialization and excludes the initial training samples. Since one sample is acquired per iteration, iteration k corresponds to $n_{0}+k$ labeled samples.

**Figure 2:**
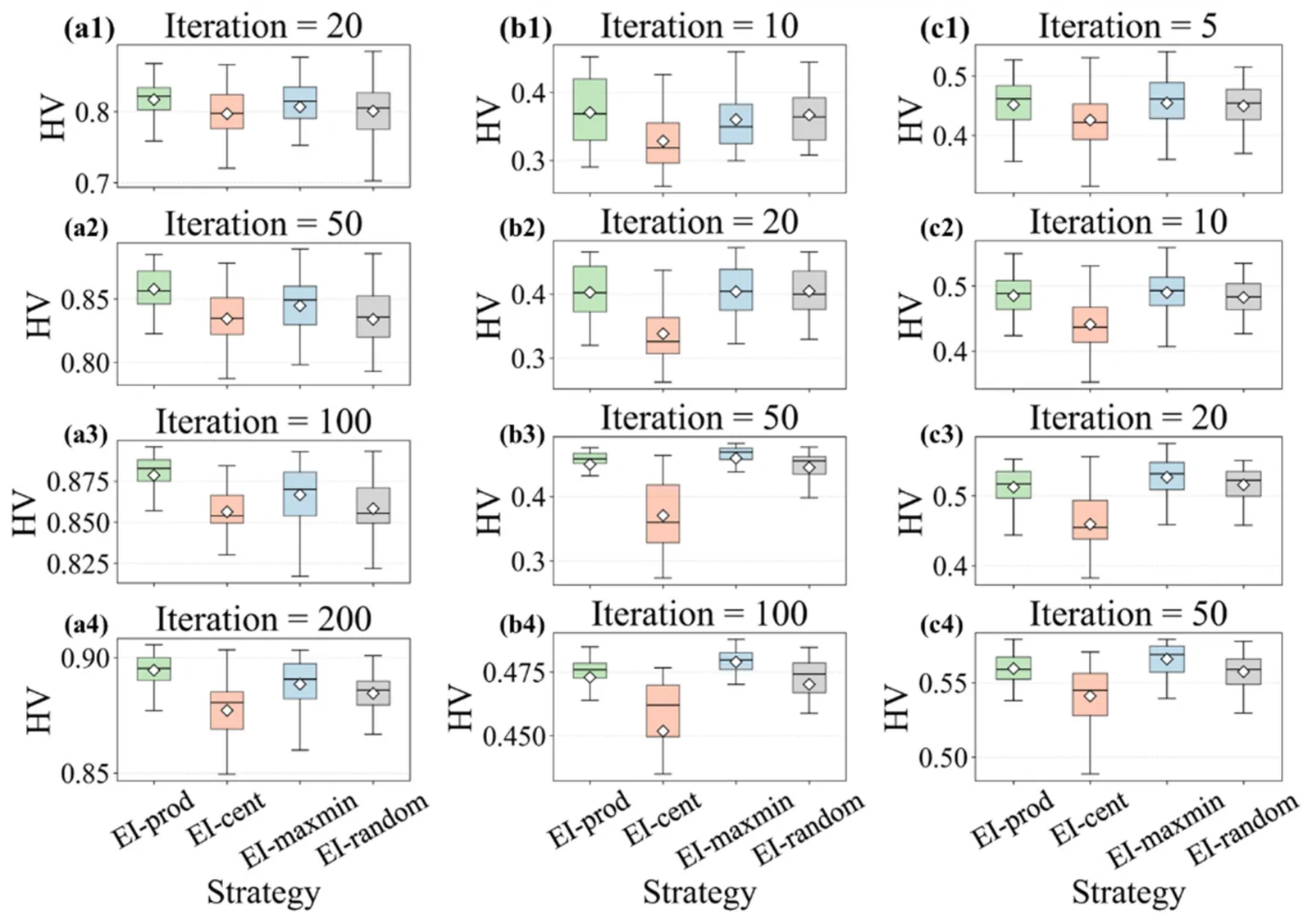
Distributions of HV obtained by EI-prod, EI-cent, EI-maxmin, and EI-random at selected active-learning iterations for the (**a1**–**a4**) Al alloy, (**b1**–**b4**) steel, and (**c1**–**c4**) Cu alloy datasets. The checkpoints are 20, 50, 100, and 200 iterations for Al; 10, 20, 50, and 100 iterations for steel; and 5, 10, 20, and 50 iterations for Cu. Each box summarizes the results of 100 paired runs at the indicated checkpoint. Boxes span the interquartile range, from the 25th to the 75th percentile; horizontal lines indicate medians, and white diamonds indicate means. Whiskers extend to the most extreme observations within 1.5 times the interquartile range from the box boundaries. Outliers beyond the whiskers are not displayed. Higher HV indicates better objective-space coverage. The checkpoints refer to acquisitions after initialization. For example, the Cu checkpoint at iteration 5 corresponds to 15 labeled samples, comprising 10 initial samples and 5 subsequently acquired samples.

Figure 1 and Figure 2 compare the HV obtained by the four acquisition strategies. HV generally increases with the sampling budget for all three datasets, confirming that successive acquisitions expand the dominated objective-space region. Nevertheless, the rate of improvement and the relative ranking of the strategies vary across datasets.

For the Al dataset, EI-prod exhibits the fastest HV growth and maintains the highest average HV over most of the active-learning process (Figure 1a). This advantage becomes more evident as the sampling budget increases. The distributions at representative iterations support the average trend: the EI-prod boxplots are progressively shifted toward higher HV values and generally retain favorable lower quartiles (Figure 2a1–a4. Thus, its performance is not driven solely by a small number of successful runs. EI-maxmin is typically the second-best strategy, whereas EI-cent and EI-random show slower improvement and less favorable late-stage distributions.

The Al results are consistent with the emphasis of EI-prod on simultaneous expected improvement across objectives. Its multiplicative score downweights candidates promising in only one objective, which may direct sampling toward jointly favorable regions. The observed HV advantage suggests that this preference is useful for the Al candidate pool; the trajectories alone do not establish a causal explanation based on Pareto geometry.

A different pattern is observed for steel. Although EI-prod remains competitive during the early stage, EI-maxmin exhibits a higher observed mean HV at larger acquisition budgets (Figure 1b). The upward shift in its HV distribution at the intermediate and final checkpoints shows that this improvement is visible across repeated runs (Figure 2b2–b4). One interpretation is that the readily accessible high-performance region can initially be identified through joint improvement, whereas continued HV expansion requires the exploration of underrepresented parts of the Pareto front. By favoring candidates distant from their nearest known Pareto point, EI-maxmin reduces redundant acquisitions and progressively fills local gaps in objective space.

The Cu dataset displays a similar transition. The strategies are relatively close at small sampling budgets, but EI-maxmin develops a clearer advantage during the intermediate and late stages (Figure 1c). Its final HV distribution is concentrated near the upper observed range (Figure 2c4), which is descriptively consistent with favorable objective-space coverage across the evaluated initializations. EI-random can remain competitive at early checkpoints because unbiased sampling may occasionally identify useful candidates in a finite search space. However, it lacks a systematic mechanism for expanding uncovered regions and consequently becomes less effective as the sampling budget increases.

EI-cent is generally less reliable than EI-prod and EI-maxmin. A large distance from the centroid does not necessarily indicate that a candidate occupies an uncovered region; it may instead lie near an already represented extreme. This limitation can lead to redundant exploration and may contribute to the lower central values or wider distributions observed for EI-cent in several panels of Figure 2.

The observed mean HV trajectories descriptively favor EI-prod for Al and EI-maxmin at larger budgets for steel and Cu. These trends are specific to the evaluated conditions and do not establish statistically significant between-strategy differences. The checkpoint distributions in Figure 2 broadly support the mean trends in Figure 1, although overlapping distributions caution against interpreting every difference as statistically established.

### 3.2. Discovery of True Pareto-Optimal Samples

**Figure 3:**
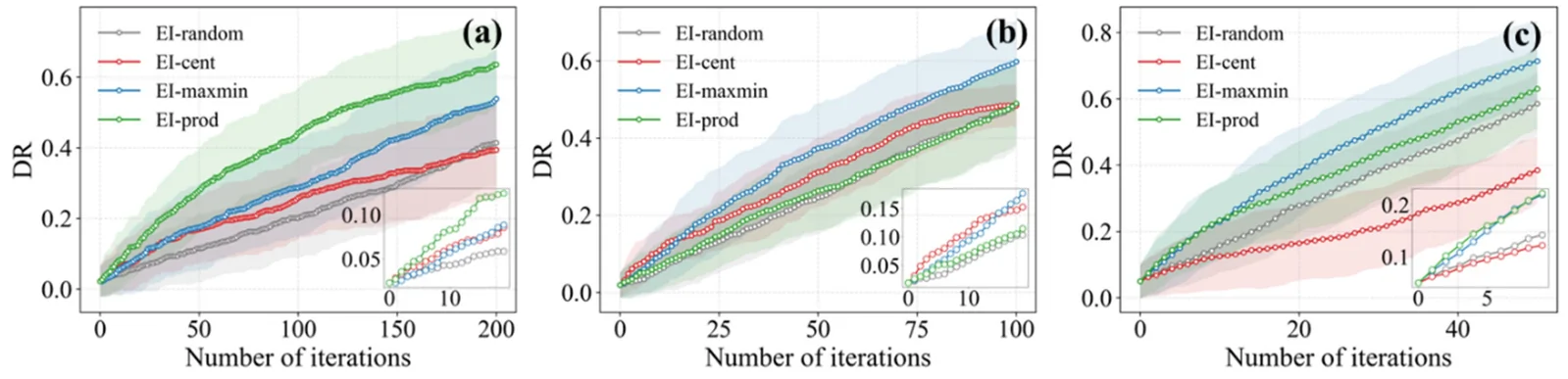
Evolution of the DR of true Pareto-optimal samples with the number of active-learning iterations for the (**a**) Al alloy, (**b**) steel, and (**c**) Cu alloy datasets. The curves represent EI-random, EI-cent, EI-maxmin, and EI-prod, while each curve represents the mean across 100 paired runs, and the shaded color-band indicates the standard deviation across these runs. Within each dataset, the four strategies share the same initial labeled set in each paired run. Insets enlarge the initial optimization stage. A higher DR indicates that a larger fraction of the global Pareto-optimal set has been identified. The horizontal axis counts acquisitions after initialization and excludes the initial training samples. Since one sample is acquired per iteration, iteration k corresponds to $n_{0}+k$ labeled samples.

**Figure 4:**
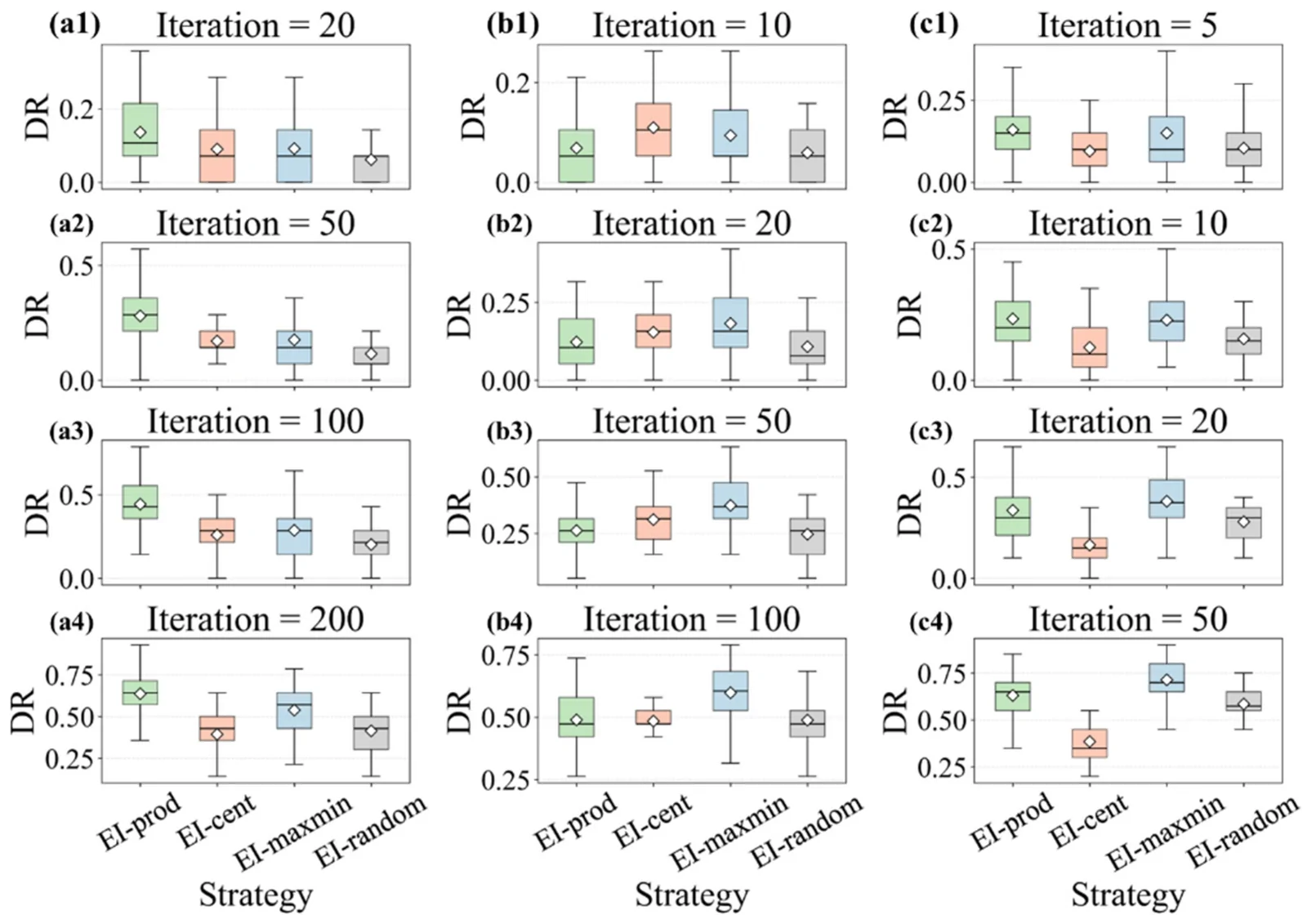
Distributions of the DR obtained by EI-prod, EI-cent, EI-maxmin, and EI-random at selected active-learning iterations for the (**a1**–**a4**) Al alloy, (**b1**–**b4**) steel, and (**c1**–**c4**) Cu alloy datasets. The checkpoints are 20, 50, 100, and 200 iterations for Al; 10, 20, 50, and 100 iterations for steel; and 5, 10, 20, and 50 iterations for Cu. Each box summarizes the results of 100 paired runs at the indicated checkpoint. Boxes span the interquartile range, from the 25th to the 75th percentile; horizontal lines indicate medians, and white diamonds indicate means. Whiskers extend to the most extreme observations within 1.5 times the interquartile range from the box boundaries. Outliers beyond the whiskers are not displayed. A higher DR indicates that a larger fraction of the true Pareto-optimal samples has been discovered. The checkpoints refer to acquisitions after initialization. For example, the Cu checkpoint at iteration 5 corresponds to 15 labeled samples, comprising 10 initial samples and 5 subsequently acquired samples.

Figure 3 and Figure 4 report the DR achieved during active learning. The results broadly agree with the HV comparison but also reveal differences between expanding the dominated region and recovering individual Pareto-optimal samples.

For Al, EI-prod produces the fastest increase in DR and retains the highest average performance over most of the sampling process (Figure 3a). Its distributions at the selected checkpoints are generally shifted toward higher values (Figure 4(a1–a4)), indicating that the strategy consistently recovers a larger fraction of the true Pareto set. EI-maxmin becomes increasingly competitive at larger budgets, whereas EI-cent and EI-random require more acquisitions to achieve comparable recovery. The consistency between HV and DR indicates that the advantage of EI-prod is not caused solely by a few strongly dominating solutions; it also corresponds to the discovery of a substantial number of true Pareto-optimal samples.

For steel, the relative ranking changes with the sampling stage (Figure 3b). EI-cent can be competitive during the early iterations because sampling far from the center of the current Pareto approximation may rapidly identify some extreme Pareto-optimal candidates. However, its subsequent mean DR increases more slowly, whereas EI-maxmin exhibits a higher observed mean DR at larger sampling budgets. The late-stage distributions of EI-maxmin (Figure 4(b3,b4)) are consistent with nearest-neighbor-based exploration being more effective for systematically recovering Pareto regions that remain unrepresented.

EI-prod also supports early discovery in steel but does not retain a clear advantage at larger budgets. Because it favors candidates with joint expected improvement, its sampling may focus on balanced high-performance regions while giving less attention to trade-off solutions. This interpretation is consistent with the later DR advantage of EI-maxmin, whose nearest-front-point distance encourages coverage of less-represented regions.

For Cu, the differences among the strategies are relatively small during the initial stage but become clearer at intermediate and late checkpoints (Figure 3c). EI-maxmin ultimately achieves the most favorable DR, as also reflected by the higher late-stage distributions in Figure 4(c3,c4). Its increasing advantage suggests that local diversity becomes important after the most readily identifiable Pareto-optimal candidates have been labeled. EI-cent remains less consistent, with some runs recovering a reasonable proportion of the true front and others exhibiting delayed discovery.

DR and HV capture different aspects of search quality: a few well-placed solutions can produce a high HV, whereas high DR requires recovery of many members of the dataset-wide Pareto set. The broadly favorable results for EI-prod on Al and EI-maxmin on steel and Cu therefore extend beyond dominated-space expansion alone. Differences between the rankings also show why neither metric should be used as a substitute for the other.

The DR distributions in Figure 4 tend to be wider at early checkpoints, consistent with sensitivity to the initial labeled set. They generally shift upward as more candidates are acquired, although lower tails persist for some strategies. The comparatively favorable late-stage distributions of EI-prod for Al and EI-maxmin for steel and Cu indicate more consistent recovery of the fixed empirical reference sets across randomized runs. This consistency concerns initialization and algorithmic variability; robustness to repeated measurement noise was not assessed.

### 3.3. Convergence and Representativeness of the Reconstructed Pareto Fronts

**Figure 5:**
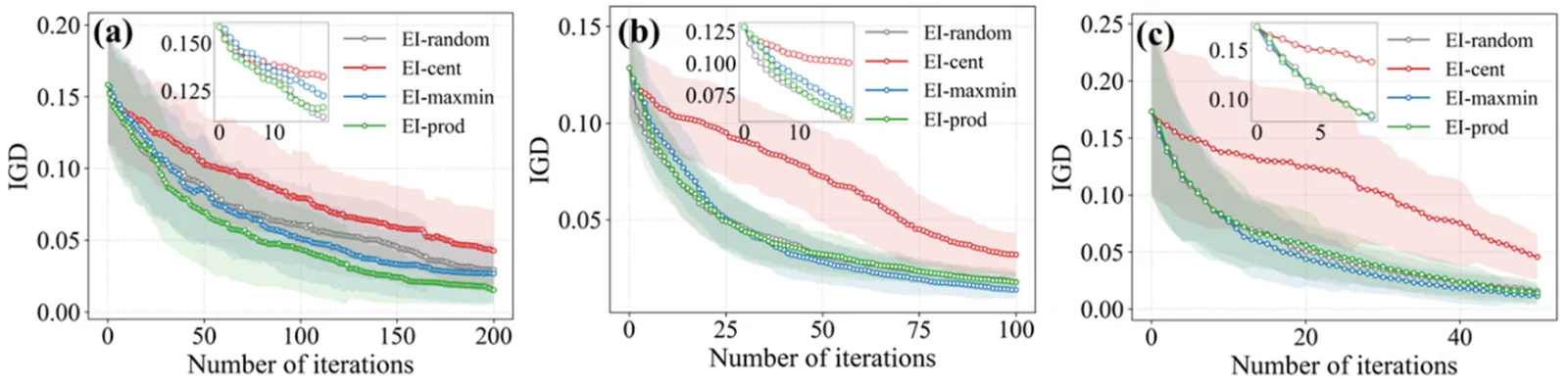
Evolution of the IGD with the number of active-learning iterations for the (**a**) Al alloy, (**b**) steel, and (**c**) Cu alloy datasets. The curves correspond to EI-random, EI-cent, EI-maxmin, and EI-prod, and each curve represents the mean across 100 paired runs; the shaded color-band indicates the standard deviation across these runs. Within each dataset, the four strategies share the same initial labeled set in each paired run. Insets enlarge the initial optimization stage. A lower IGD indicates closer convergence to and better representation of the true Pareto front. The horizontal axis counts acquisitions after initialization and excludes the initial training samples. Since one sample is acquired per iteration, iteration k corresponds to $n_{0}+k$ labeled samples.

**Figure 6:**
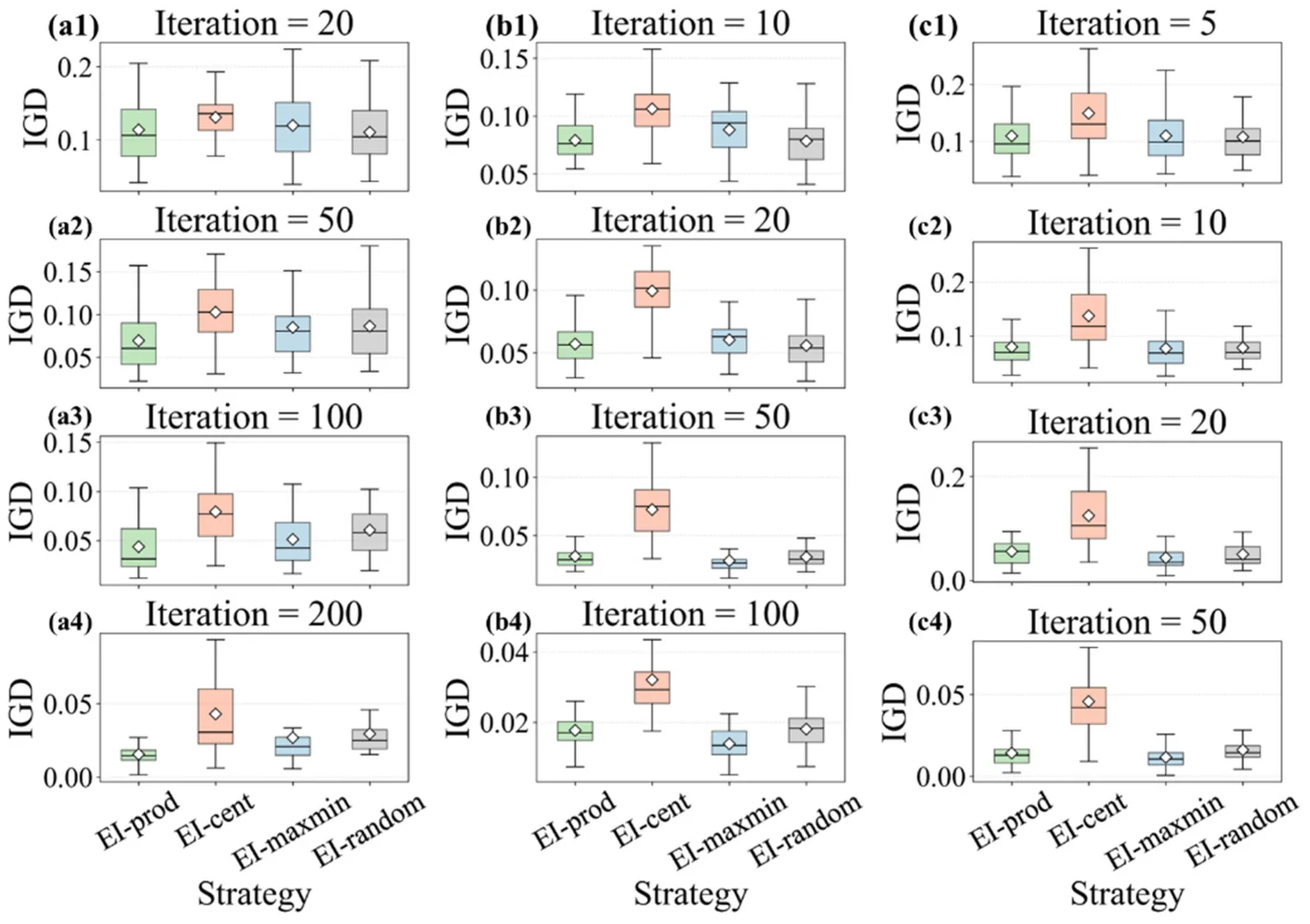
Distributions of IGD obtained by EI-prod, EI-cent, EI-maxmin, and EI-random at selected active-learning iterations for the (**a1**–**a4**) Al alloy, (**b1**–**b4**) steel, and (**c1**–**c4**) Cu alloy datasets. The checkpoints are 20, 50, 100, and 200 iterations for Al; 10, 20, 50, and 100 iterations for steel; and 5, 10, 20, and 50 iterations for Cu. Each box summarizes the results of 100 paired runs at the indicated checkpoint. Boxes span the interquartile range, from the 25th to the 75th percentile; horizontal lines indicate medians, and white diamonds indicate means. Whiskers extend to the most extreme observations within 1.5 times the interquartile range from the box boundaries. Outliers beyond the whiskers are not displayed. A lower IGD indicates closer convergence to and better representation of the true Pareto front. The checkpoints refer to acquisitions after initialization. For example, the Cu checkpoint at iteration 5 corresponds to 15 labeled samples, comprising 10 initial samples and 5 subsequently acquired samples.

Figure 5 and Figure 6 evaluate the reconstructed fronts using IGD. IGD decreases with the number of acquisitions in all three datasets, showing progressive convergence toward the true Pareto fronts. The strategies nevertheless differ in both convergence rate and run-to-run variability.

For Al, EI-prod provides the most rapid and sustained reduction in IGD (Figure 5a). At the early checkpoint, the distributions of the strategies overlap substantially, indicating sensitivity to the initial labeled set (Figure 6(a1)). With further acquisitions, the EI-prod distribution shifts downward more rapidly than those of the competing strategies. At the final checkpoint (Figure 6(a4)), it exhibits the lowest central IGD and a comparatively concentrated distribution. EI-maxmin generally ranks second, whereas EI-cent retains a higher IGD and a more pronounced upper tail.

The IGD results complement the corresponding HV and DR results. On Al, EI-prod expands the dominated region and recovers many true Pareto-optimal samples while also producing a closer approximation to the full reference front. This agreement across metrics argues against interpreting its HV advantage as the result of only a few isolated solutions.

For steel, EI-prod and EI-random have comparable observed mean IGD values during the early stage, whereas EI-maxmin exhibits the lowest observed mean IGD at intermediate and larger budgets (Figure 5b). By the final checkpoint, EI-maxmin has the lowest IGD distribution (Figure 6(b4)), whereas EI-prod and EI-random remain slightly higher and EI-cent is clearly inferior. The relatively compact late-stage distribution of EI-maxmin describes its observed run-to-run variability; it does not independently establish a statistically significant convergence advantage.

This transition is consistent with the nearest-front-point distance used by EI-maxmin. After an initial front approximation has been established, sampling less-represented regions may improve its coverage and reduce IGD. The metric trajectories support this interpretation but do not isolate the acquisition mechanism from dataset effects.

A related trend appears for Cu. The differences among EI-prod, EI-maxmin, and EI-random are limited at the earliest checkpoint, whereas EI-cent generally produces higher and more dispersed IGD values (Figure 6(c1)). EI-maxmin subsequently develops the lowest IGD and maintains this advantage through the final stage (Figure 5c and Figure 6(c3,c4)). Its late-stage distribution is both low and relatively narrow. In contrast, the persistent upper range of EI-cent indicates that substantial portions of the true front remain insufficiently represented in some runs.

Comparing DR with IGD helps explain why recovering several Pareto-optimal samples need not yield a representative front. Samples clustered in one region can increase DR while leaving distant sections poorly covered and IGD relatively high. This distinction is especially relevant to EI-cent on steel: distance from a single centroid can favor extremes without ensuring coverage between them. In contrast, the local distance term in EI-maxmin is consistent with its better late-stage IGD.

Taken together, HV, DR, and IGD favor EI-prod for front reconstruction on Al and EI-maxmin at larger budgets on steel and Cu. Early distributions sometimes overlap, so the practical advantage depends on the sampling budget as well as the dataset.

### 3.4. Discovery and Sample Efficiency of Balanced High-Performance Solutions

**Figure 7:**
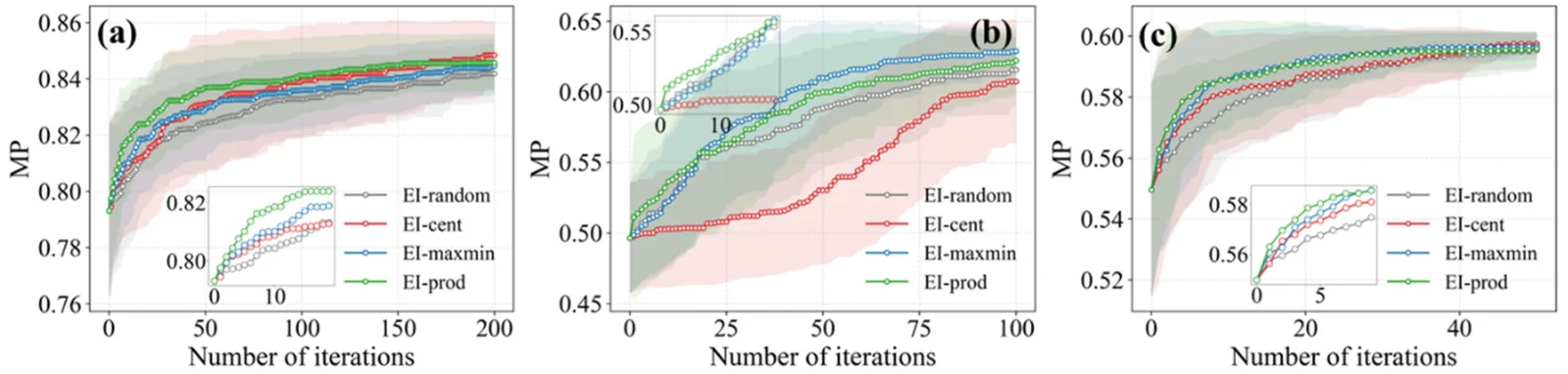
Evolution of the MP, calculated as the maximum geometric mean of the normalized objective values among the labeled samples, for the (**a**) Al alloy, (**b**) steel, and (**c**) Cu alloy datasets. The curves correspond to EI-random, EI-cent, EI-maxmin, and EI-prod, and each curve represents the mean across 100 paired runs, and the shaded band indicates the standard deviation across these runs. Within each dataset, the four strategies share the same initial labeled set in each paired run. Insets enlarge the initial active-learning stage. A higher MP indicates the discovery of a material with a better balanced combination of objective properties. The horizontal axis counts acquisitions after initialization and excludes the initial training samples. Since one sample is acquired per iteration, iteration k corresponds to $n_{0}+k$ labeled samples.

**Figure 8:**
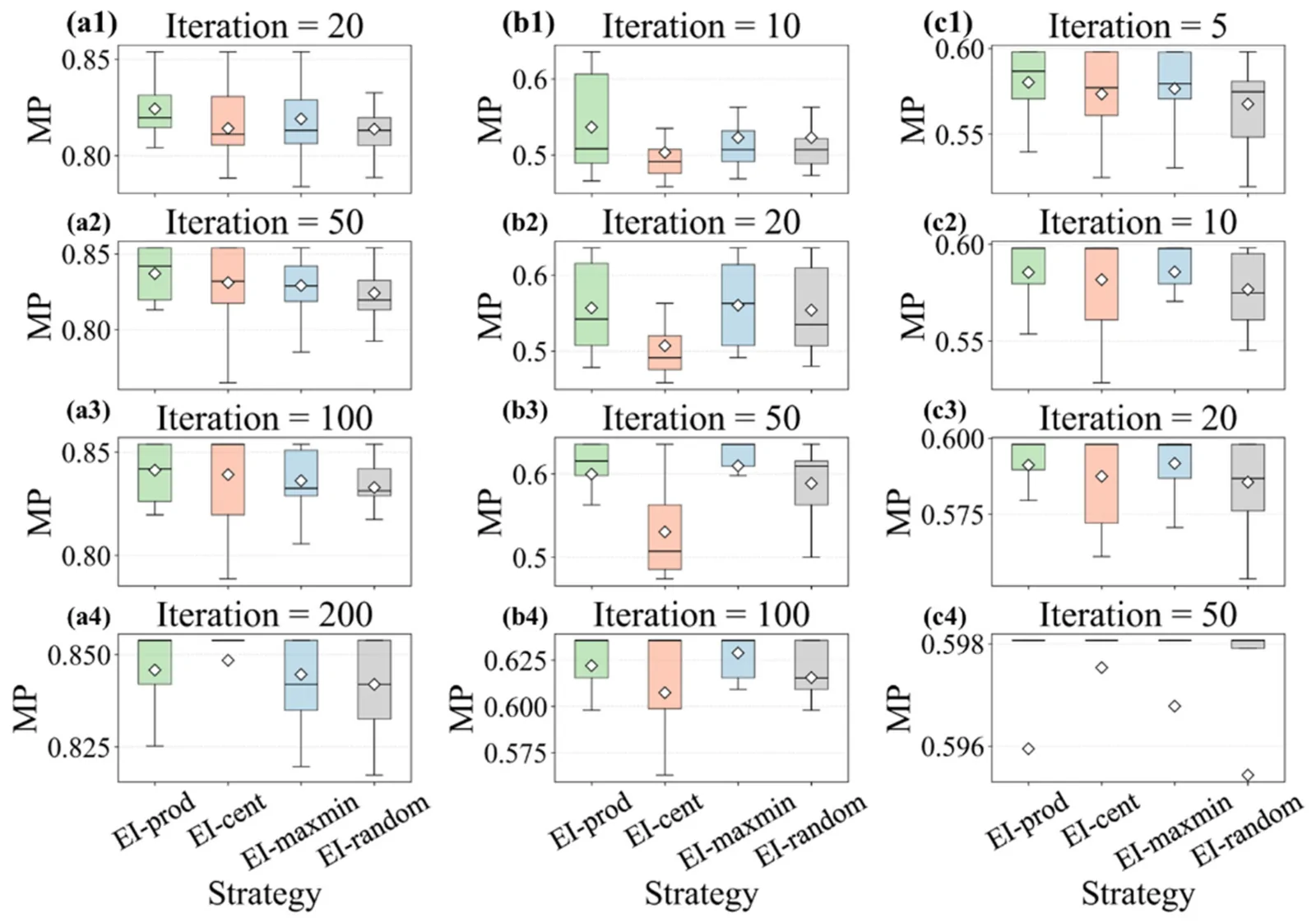
Distributions of the MP obtained by EI-prod, EI-cent, EI-maxmin, and EI-random at selected active-learning iterations for the (**a1**–**a4**) Al alloy, (**b1**–**b4**) steel, and (**c1**–**c4**) Cu alloy datasets. The checkpoints are 20, 50, 100, and 200 iterations for Al; 10, 20, 50, and 100 iterations for steel; and 5, 10, 20, and 50 iterations for Cu. Each box summarizes the results of 100 paired runs at the indicated checkpoint. Boxes span the interquartile range, from the 25th to the 75th percentile; horizontal lines indicate medians, and white diamonds indicate means. Whiskers extend to the most extreme observations within 1.5 times the interquartile range from the box boundaries. Outliers beyond the whiskers are not displayed. A higher MP indicates discovery of a solution with a better-balanced combination of normalized objective values. The checkpoints refer to acquisitions after initialization. For example, the Cu checkpoint at iteration 5 corresponds to 15 labeled samples, comprising 10 initial samples and 5 subsequently acquired samples.

**Figure 9:**
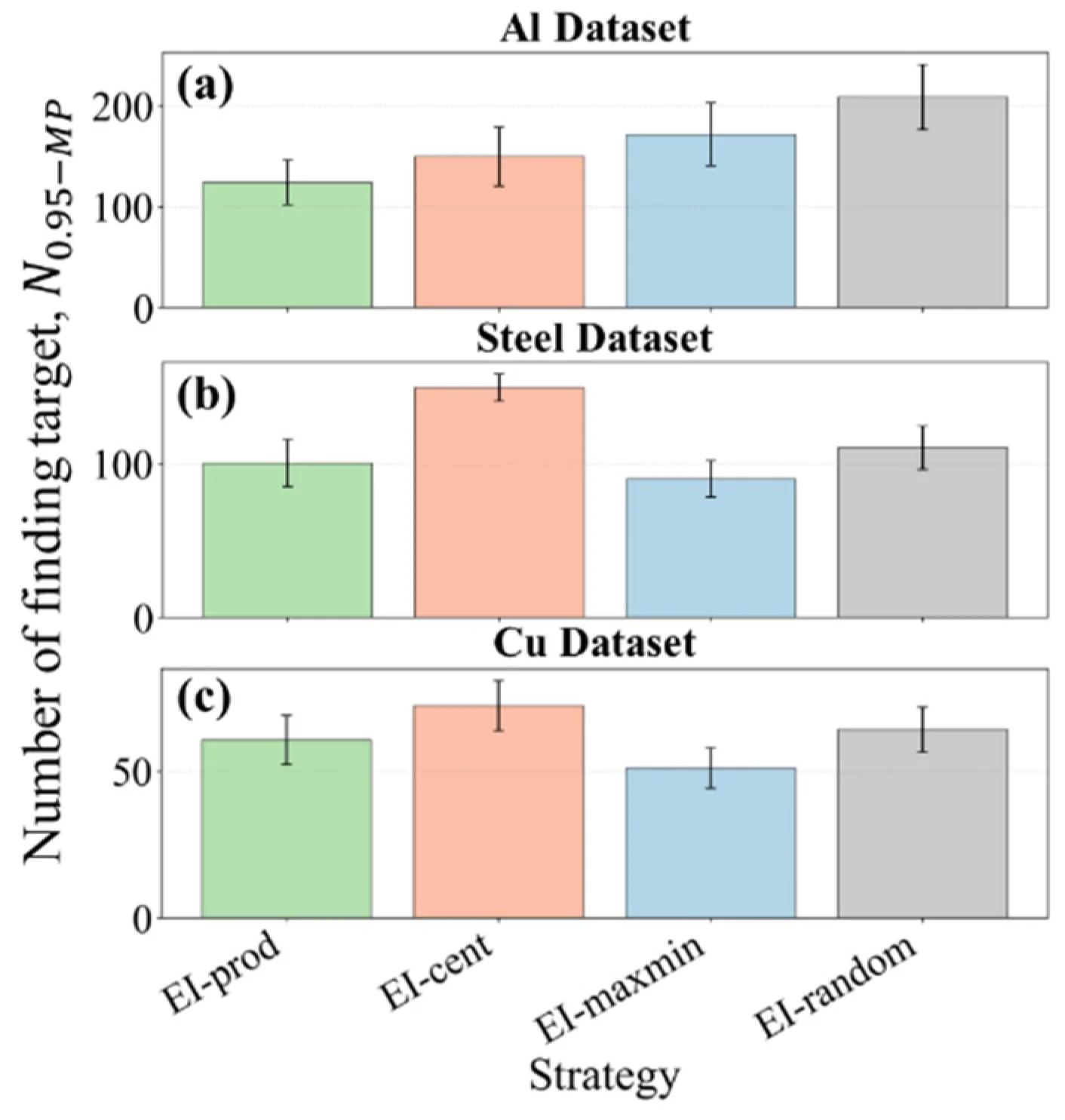
Total number of labeled samples required by EI-prod, EI-cent, EI-maxmin, and EI-random to reach 95% of the reference MP score, N_0_._95_ − MP, for the (**a**) Al alloy, (**b**) steel, and (**c**) Cu alloy datasets. Bars represent the mean total number of labeled samples required to reach the target, calculated only over successful runs, and error bars indicate ±1 standard deviation among those runs. The total labeled-sample count includes the initial training samples. Runs that do not reach the target within the maximum budget are excluded from these conditional summaries, and their frequency is reflected in the attainment rates reported in Figure 10. Lower values correspond to greater sample efficiency. The reported budgets include the initial training samples. The corresponding number of acquisitions after initialization is obtained by subtracting $n_{0}$ from the total labeled-sample count.

Figure 7 and Figure 8 compare MP, the best geometric-mean-based balanced-performance score among acquired samples, while Figure 9 reports the iterations needed to reach 95% of its dataset-wide reference value. Unlike HV, DR, and IGD, MP can be maximized by discovering a single suitable candidate without reconstructing the entire Pareto front.

For Al, EI-prod achieves the highest MP during the early and intermediate stages (Figure 7a). Its distributions are generally shifted upward and exhibit favorable lower quartiles (Figure 8(a1–a3)), indicating reliable early discovery of high-quality compromise solutions. EI-cent and EI-maxmin occasionally obtain comparable MP values, but their distributions extend farther toward lower values. As the budget approaches 200 iterations, all four strategies converge toward a similar upper MP level (Figure 8(a4)), and the differences in their final central values become small.

Similar late-stage MP values do not erase the earlier advantage of EI-prod on Al. EI-prod multiplies the expected improvements of the individual objectives, whereas MP rewards candidates that achieve simultaneously high normalized values across the objectives through a geometric mean. These two quantities are mathematically different and are calculated from different information: EI-prod uses model predictions and predictive uncertainty during candidate selection, while MP is evaluated from the measured objective values of the labeled samples. Nevertheless, their shared emphasis on simultaneous multi-objective improvement may create a favorable structural alignment for EI-prod when MP is used as the evaluation metric. This possible alignment may contribute to the early MP trend observed for Al, but it does not establish a causal relationship or imply that EI-prod is generally optimal for balanced-performance discovery. The inclusion of HV, DR, and IGD provides complementary evidence because these metrics evaluate objective-space coverage, Pareto-front discovery, and front representation rather than only balanced performance.

For steel, EI-prod generates some high-MP outcomes during the early stage, although its distribution is relatively broad (Figure 8(b1)). EI-cent has the lowest central performance and frequently fails to identify an improved compromise solution within a small budget. As active learning proceeds, the observed MP distributions become descriptively favorable for EI-maxmin. At 50 and 100 iterations, it exhibits the highest or most favorable MP distributions (Figure 8(b3,b4)), with less pronounced lower tails than EI-cent and EI-random.

**Figure 10:**
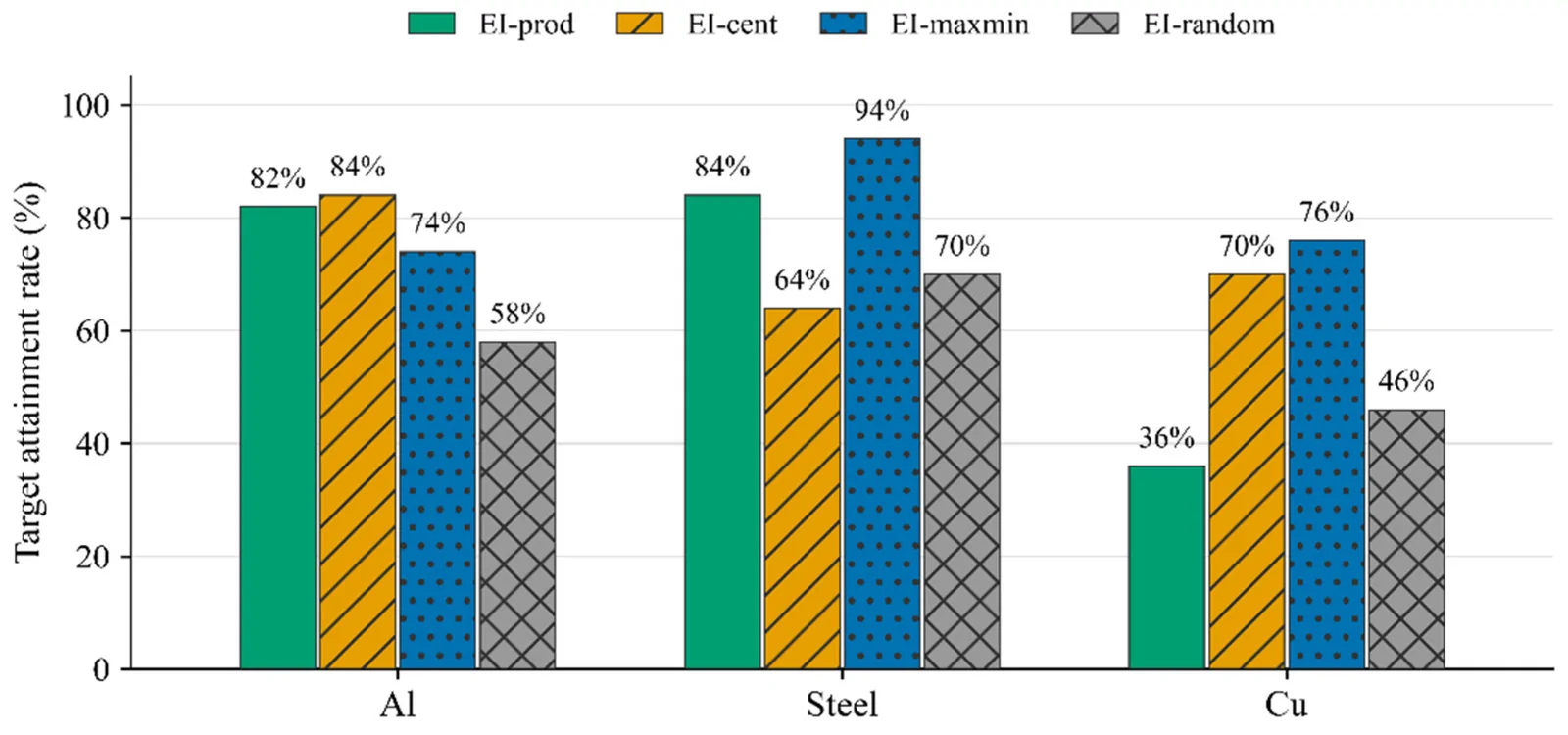
Target attainment rates of the four acquisition strategies within the prescribed maximum budget for the Al, steel, and Cu datasets. The attainment rate is the percentage of the 100 trials that reached 95% of the reference MP before or at the budget limit. Unsuccessful trials are reported as failures and are not assigned an observed target-reaching time.

For steel, EI-maxmin reaches 95% of the reference MP after approximately 90 iterations, followed by EI-prod at about 100, EI-random at about 110, and EI-cent at about 150 (Figure 9b). The difference between the reported means is descriptive, and the plotted error bars do not establish whether the between-strategy difference is statistically significant. Both appear more efficient than EI-cent in this comparison, consistent with the value of exploring less-represented Pareto regions. It is noted that differences between these conditional means should not be interpreted as paired estimates of sampling-budget savings. The attainment rates in Figure 10 provide complementary information about reaching the target within the available budget.

For Cu, EI-prod has the highest or among the highest MP during the earliest iterations, while EI-maxmin remains highly competitive (Figure 7c and Figure 8(c1,c2)). The distributions of all strategies subsequently approach a common upper limit. At 50 iterations, most medians are nearly identical and several boxes collapse close to the maximum value (Figure 8(c4)). The lower means of some strategies are mainly caused by a small number of runs in which discovery of the best candidate is delayed.

This ceiling effect limits the distinction between strategies based on final MP alone. Figure 9c provides an additional descriptive comparison, with EI-maxmin showing the lowest observed conditional mean target-reaching budget. This result should be considered together with the attainment rates in Figure 10 and does not, by itself, establish a paired sampling-efficiency gain across all runs.

To account for unsuccessful trials, Figure 10 reports the fraction of the 100 trials that reached the 95% MP target within the maximum budget. For Al, the observed attainment rates were 82% for EI-prod, 84% for EI-cent, 74% for EI-maxmin, and 58% for EI-random. For steel, the corresponding rates were 84%, 64%, 94%, and 70%, respectively. For Cu, the rates were 36%, 70%, 76%, and 46%, respectively. These results show that the strategy with the lowest conditional target-reaching budget is not necessarily the strategy with the highest probability of reaching the target within the available budget. Figure 9 and Figure 10 therefore provide complementary information: Figure 9 summarizes the target-reaching budget among successful trials, whereas Figure 10 summarizes the probability of success within the prescribed budget.

The MP results distinguish discovery of a balanced solution from reconstruction of a Pareto front. A single candidate can yield a high MP, allowing EI-cent and EI-random eventually to attain values similar to those of EI-prod and EI-maxmin even if their HV, DR, or IGD remain less favorable. Front-level metrics instead depend on multiple well-placed samples. Comparable final MP values should therefore not be taken as evidence of comparable overall search performance.

Across Figure 7, Figure 8 and Figure 9, the observed trends favor EI-prod for early balanced-candidate discovery on Al and EI-maxmin at larger budgets on steel and Cu under the evaluated conditions. These differences occur alongside variations in dataset size, composition, objective trade-offs, and reference-front geometry. Although the distance term in EI-maxmin provides a motivation for examining its relationship with front geometry, the present comparisons do not establish geometry as the cause of the observed rankings. The practical guidance is therefore based on the observed performance for the intended optimization outcome and budget, without prescribing an acquisition strategy solely from material family or front shape.

The findings above offer a metric-aware basis for choosing acquisition functions in multi-objective materials screening. However, the present study is a retrospective simulation on finite datasets with known objective values. In a prospective campaign, the selected candidates may be unavailable or infeasible, measured properties may contain experimental noise, failed experiments may occur, and different evaluations may have different costs and durations. The present results therefore support the comparison of acquisition preferences under a common candidate-pool protocol, but they should not be interpreted as direct predictions of experimental success or cost reduction. Prospective validation should combine the acquisition functions with feasibility constraints, experimental uncertainty models, cost-aware budgets, and periodic model recalibration.

## 4. Conclusions

We compared EI-random, EI-cent, EI-maxmin, and EI-prod on three materials datasets (Al, steel, and Cu) using complementary metrics of dominated-space coverage (HV), true Pareto-sample discovery (DR), front representation (IGD), and balanced-candidate discovery (MP). The following findings summarize the observed mean trajectories, checkpoint distributions, and conditional target-reaching budgets. They provide descriptive comparisons within the evaluated benchmark, without establishing statistically supported paired sampling-efficiency gains.

(1) EI-prod and EI-maxmin generally compare favorably with EI-random, while EI-cent offers limited and inconsistent gains. On Al, EI-prod provides the strongest HV, DR, IGD, and MP trends and reaches the 95%-MP target in approximately 120–125 iterations.
(2) At intermediate and larger budgets, EI-maxmin gives the most favorable front-recovery and convergence trends on steel and Cu. It reaches the 95%-MP target after approximately 90 iterations on steel and 50 on Cu in these retrospective simulations.
(3) Similar final MP values do not imply equivalent front-level performance: HV, DR, and IGD additionally assess coverage and representation of Pareto-optimal trade-offs.
(4) Among the four investigated strategies, no single strategy consistently leads across all three datasets and budgets. Within this comparison, EI-prod merits consideration for early joint improvement, whereas EI-maxmin merits consideration for broader Pareto-front exploration. These observations provide guidance within the evaluated strategy set; their extension to EHVI and other acquisition functions remains to be assessed.
(5) The target attainment rates further show that favorable mean trajectories do not necessarily imply the highest probability of reaching the target within a fixed budget. For example, EI-cent and EI-prod attained the Al target in 84% and 82% of trials, respectively, whereas EI-maxmin had the highest observed attainment rates on steel and Cu.

These findings provide dataset- and budget-specific guidance for the four investigated strategies. The present comparison does not separate the effects of sample size, material composition, objective definitions, and Pareto-front geometry. Controlled benchmarks that vary front geometry while holding other factors fixed would help determine which geometric characteristics contribute to the observed strategy differences. The rankings are also conditional on the fixed full-dataset objective normalization used in this retrospective benchmark. Their transfer to prospective sampling with initially unknown objective ranges requires assessment under scaling based on sequentially available information. The comparisons also depend on the adopted Gaussian predictive approximation, which uses bootstrap ensemble spread as an uncertainty proxy and does not explicitly model cross-objective predictive covariance. Uncertainty calibration and covariance-aware modeling would help assess the stability of the observed rankings, particularly for materials with pronounced property trade-offs.

## Figures and Tables

**Table 1:** Description of the three datasets used in the present study.

Dataset	Sample Size	Total Number of Features	Input Descriptors	Optimal Objects	Scope of the Objects	Size of the True Pareto-Optimal Set
Al	1043	16	Zn, Mg, Cu, Cr, Mn, and Zr contents; primary and secondary solution-treatment temperatures and times; primary, secondary, and tertiary aging temperatures and times.	UTS, EA	UTS: 312–722 EA: 3.0–18.1	14
Steel	437	15	NT, THT, DT, Dt, QmT, TT, C, Si, Mn, Cr, Cu, Mo, Ratio, dA, and dB, retaining the original dataset field names.	UTS, EA	UTS: 455–1756 EA: 0.2–40.0	19
Cu	190	10	Fe, P, Ni, Si, Mg, Zn, Sn, Cr, Zr, and Re contents	UTS, EC	UTS: 340–1155.8 EC: 12–95	20

**Table 2:** Selected numerical results at fixed evaluation budgets, reported as mean ± standard deviation across 100 paired runs.

Dataset	*N*	Strategy	HV ↑	DR ↑	IGD ↓
Al	422	EI-prod	0.8945 ± 0.0073	0.6357 ± 0.1235	0.0155 ± 0.0086
Al	422	EI-cent	0.8770 ± 0.0127	0.3943 ± 0.1363	0.0428 ± 0.0283
Al	422	EI-maxmin	0.8885 ± 0.0110	0.5386 ± 0.1393	0.0267 ± 0.0211
Al	422	EI-random	0.8845 ± 0.0096	0.4143 ± 0.1199	0.0294 ± 0.0156
Steel	210	EI-prod	0.4728 ± 0.0125	0.4895 ± 0.1075	0.0177 ± 0.0052
Steel	210	EI-cent	0.4517 ± 0.0288	0.4842 ± 0.0542	0.0321 ± 0.0114
Steel	210	EI-maxmin	0.4789 ± 0.0043	0.5979 ± 0.1057	0.0139 ± 0.0044
Steel	210	EI-random	0.4701 ± 0.0137	0.4884 ± 0.1115	0.0181 ± 0.0057
Cu	110	EI-prod	0.5595 ± 0.0113	0.6300 ± 0.1212	0.0141 ± 0.0080
Cu	110	EI-cent	0.5410 ± 0.0185	0.3850 ± 0.1011	0.0456 ± 0.0199
Cu	110	EI-maxmin	0.5658 ± 0.0109	0.7140 ± 0.1208	0.0117 ± 0.0078
Cu	110	EI-random	0.5573 ± 0.0120	0.5850 ± 0.0954	0.0161 ± 0.0069

Note: *N* denotes the total number of labeled samples, including initialization. Standard deviations are calculated across runs using the sample standard deviation with a denominator of n−1, where  n=100. Higher (labelled by **↑**) HV and DR and lower (labelled by **↓**) IGD indicate more favorable performance. Budgets are identical across strategies within each dataset but differ between datasets.

## Data Availability

The original contributions presented in this study are included in the article. Further inquiries can be directed to the corresponding authors.
